# Supplementary material for: Features of Patients Receiving Extracorporeal Membrane Oxygenation Relative to Cardiogenic Shock Onset: A Single-Centre Experience
Source: Medicina (Kaunas). 2021 Aug 27;57(9):886. doi: 10.3390/medicina57090886 (PMC8465743; doi:10.3390/medicina57090886)
Supplement: Supplementary file 1 [file medicina-57-00886-s001.zip › Supplementary_TableS1.pdf]

Supplementary Table S1. The factors associated with bailout ECMO insertion, and found that advanced age (≥ 70 years), preadmission CS, absence of hypertension, and starting CRP within 9 min of the MI were beneficial; however, the benefits were not statistically significant

	OR	lower	upper	p
**≥70**	**1.071429**	**0.1498046**	**9.295385**	**0.95**
**<70**	**0.6346154**	**0.1621109**	**2.383080**	**0.50**
**Male**	**0.8333333**	**0.2604926**	**2.652820**	**0.76**
**prAD**	**1.625000**	**0.4124064**	**6.565729**	**0.49**
**pstAD**	**0.2807018**	**0.03196543**	**1.998874**	**0.21**
**CPR**	**0.8941176**	**0.1933593**	**3.919993**	**0.88**
**without CPR**	**0.2**	**0.02228416**	**1.288123**	**0.11**
**HTN**	**0.2755102**	**0.0487994**	**1.270656**	**0.11**
**Without HTN**	**1.846154**	**0.3918400**	**10.333493**	**0.45**
**DM**	**0.480000**	**0.07789652**	**2.574874**	**0.40**
**without DM**	**0.9176471**	**0.2263965**	**3.821796**	**0.90**
**PCI**	**0.75**	**0.02249796**	**24.66442**	**0.86**
	**0.6956522**	**0.2191687**	**2.170015**	**0.53**
**CPR within 9min**	**1.222222**	**0.1324763**	**9.559886**	**0.85**
**PCI to LM or LAD**	**0.6666667**	**0.1461941**	**2.786112**	**0.58**
