# Supplementary figures and images for: Features of Patients Receiving Extracorporeal Membrane Oxygenation Relative to Cardiogenic Shock Onset: A Single-Centre Experience
Source: Medicina (Kaunas). 2021 Aug 27;57(9):886. doi: 10.3390/medicina57090886 (PMC8465743; doi:10.3390/medicina57090886)

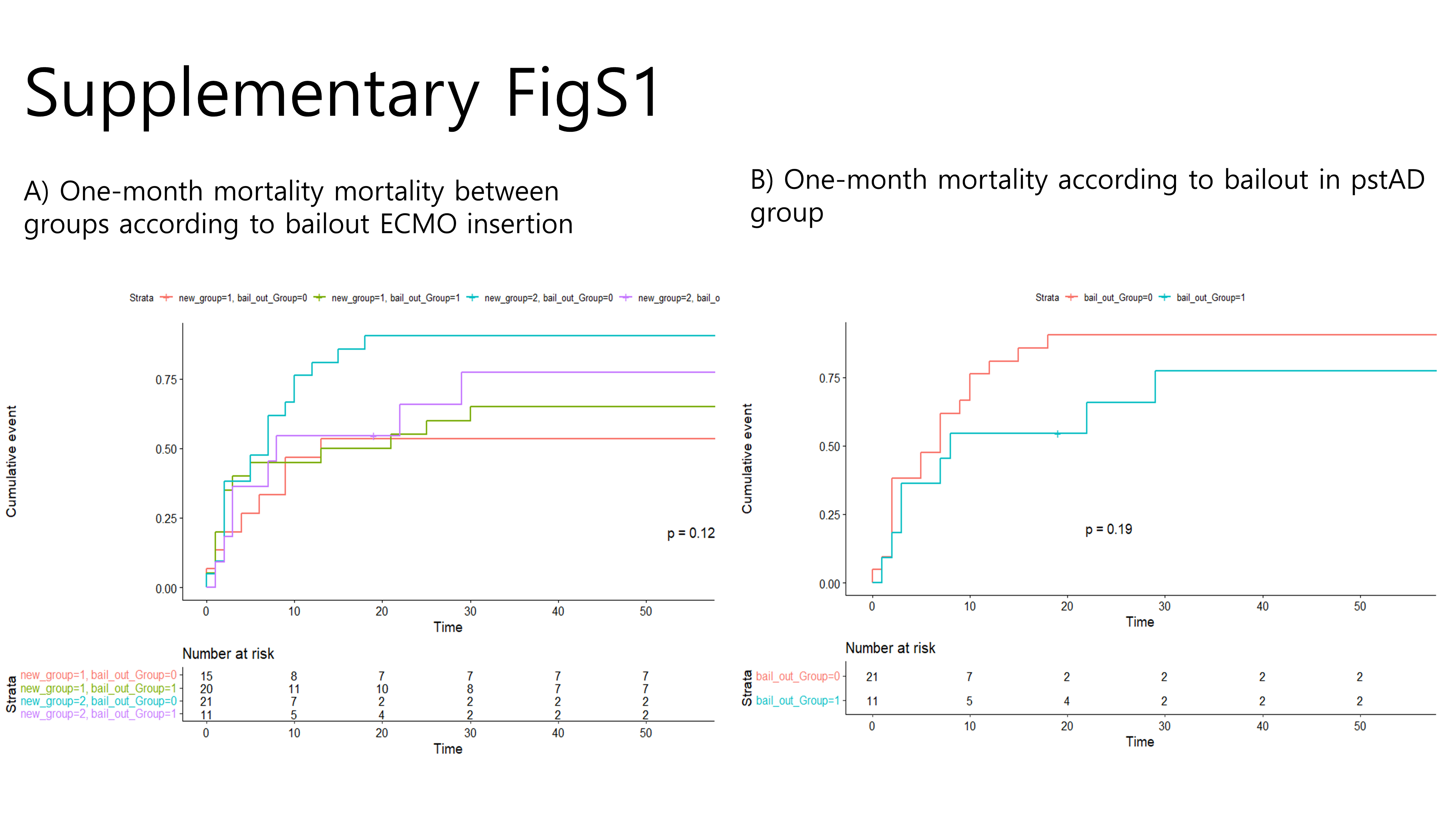

Supplement: Supplementary file 1 [file medicina-57-00886-s001.zip › Supplementary_FigS1.TIF]
